# Resolution of catatonia after propofol anaesthesia

**DOI:** 10.1177/10398562251330046

**Published:** 2025-04-16

**Authors:** Luke Lawrence Viglione, Ralf Ilchef

**Affiliations:** Lismore, NSW; Sydney, NSW

Dear Editor,

A 66-year-old man was brought to the emergency department after standing motionless and non-responsive for four hours. For the preceding week, he had complained of ‘stress’ and insomnia and had reduced food/fluid intake. On examination, he was stuporous, mute and demonstrated waxy flexibility.

The patient had had one previous psychiatric admission for psychotic depression. He had not otherwise been prescribed psychotropic medication or seen a psychiatrist. There was no relevant substance use, medical history or family history.

Given his poor oral intake and the need for organic workup, the patient was admitted under neurology. The following morning the patient’s mental state was unchanged. He underwent lumbar puncture and cerebral MRI under general anaesthetic, consisting of intravenous propofol 120 mg and inhaled sevoflurane.

In recovery, there was a marked improvement in the patient’s mental state. He was no longer mute or stuporous but demonstrated psychomotor retardation, bradyphrenia, latency of response, thought blocking and perplexed affect. He described somatic delusions and derogatory auditory hallucinations. He was oriented, attentive and with intact memory. Results of the MRI and CSF examination were unremarkable. Hence, he was transferred to the mental health unit with a provisional diagnosis of major depressive episode with catatonic and psychotic features. He was commenced on lorazepam, venlafaxine and olanzapine, and his condition eventually remitted without requiring electroconvulsive therapy.

The characteristic motor signs seen in catatonia are associated with GABA hypoactivity and glutamate overactivity, particularly implicating basal ganglia and supplementary motor cortex neural circuitry.^1^ Lorazepam, considered first-line medication for treatment of catatonia, enhances the effect of GABA on GABA-A receptors. Propofol likewise enhances GABA-ergic transmission, though at a different GABA-A subunit. It also inhibits excitatory NMDA receptors, hence attenuating the excitatory effects of glutamate.^2^ Furthermore, research suggests propofol possesses neuroprotective properties.^2^ These observations suggest that the marked reduction of our patient’s catatonic symptoms was more than coincidental.

We found three other reports of serendipitous catatonia resolution after propofol administration,^3–5^ one of which^5^ concluded that prolonged deep propofol sedation, though impractical to trial clinically, might present a valid ‘rescue treatment’ in selected cases of treatment-resistant catatonia. These clinical and psychopharmacological observations may lend weight to the selection of propofol as anaesthetic agent of choice in electroconvulsive therapy when catatonia is present.
